# Diagnostic Value of lncRNA XIST in Saliva for Early Peri-Implantitis

**DOI:** 10.3290/j.ohpd.b5656312

**Published:** 2024-08-06

**Authors:** Mei Chai, Jinzhong Zhang, Qianjiao Meng, Andong Liu

**Affiliations:** a Associate Professor, Anhui Medical College, Hefei, Anhui, China. Project development, data management, data analysis, wrote and edited the manuscript, read and approved the final manuscript.; b Resident Physician, Department of Stomatology, The First Affiliated Hospital of Anhui Medical University, Hefei, Anhui, China. Data management, data analysis and manuscript writing, read and approved the final manuscript.; c Associate Professor, Anhui Medical College, Hefei, Anhui, China. Data acquisition and data analysis, read and approved the final manuscript.; d Deputy Chief Physician, Department of Stomatology, Anhui No.2 Provincial People’s Hospital, Hefei, Anhui, China. Data acquisition and data analysis, read and approved the final manuscript.

**Keywords:** diagnosis, miR-150-5p peri-implantitis, periodontal indicators, XIST

## Abstract

**Purpose:**

To analyse the relative expression and diagnostic potential of lncRNA XIST (XIST) in peri-implantitis, and explore the related mechanism of XIST in peri-implantitis.

**Materials and Methods:**

XIST expression in saliva of patients with peri-implantitis was detected by qRT-PCR. The diagnostic significance of XIST in peri-implantitis was assessed by ROC curve. Clinical indicators of the included patients were collected and the correlation between XIST levels and peri-implant indicators was determined by Pearson correlation analysis. Bioinformatic prediction and luciferase reporter assay confirmed the targeting relationship of XIST with downstream factors.

**Results:**

Salivary XIST levels were obviously higher in patients with peri-implantitis than in the healthy control group, and the AUC value for identifying patients was 0.8742 with a sensitivity and specificity of 83.5% and 81.4%. Patients in the peri-implantitis group had higher levels of plaque index (PLI), sulcus bleeding index (SBI) and probing depth (PD) than those in the healthy control group, and the expression of XIST was positively correlated with PLI, SBI, and PD levels. In addition, miR-150-5p was confirmed to be a potential downstream target of XIST.

**Conclusion:**

XIST was overexpressed in the saliva of patients with peri-implantitis and correlated with the severity of the disease. XIST has high diagnostic significance for detecting peri-implantitis.

Implant therapy is a commonly used method of oral restoration in clinical practice, which is suitable for patients with missing teeth.^35^ Peri-implantitis refers to the inflammatory reaction of the tissues around an implant, which is one of the main causes of implant treatment failure.^5,31^ Reportedly, the occurrence of peri-implantitis among patients undergoing implant restoration is 15% to 30%.^4^ In addition, factors related to bone-tissue metabolism can affect the treatment outcome of peri-implantitis in recent studies.^12^ Generally, peri-implantitis is caused by bacterial infection. However, allergic reactions and microbial infections may also be the trigger causes,^19,28^ and factors such as long-term smoking and dietary habits also increase the risk of disease.^7^ Patients often present with redness, swelling and pain in the tissues around the implant, accompanied by oral malodor and mucosal bleeding, and may also experience pain and loosening of the implant.^18^ In worse cases, implants may even need to be removed.^22^ In order to prevent the occurrence of peri-implantitis, in addition to maintaining oral hygiene and regular professional examinations, early detection of peri-implant mucositis and intervention treatment are the primary measures to reduce the risk of peri-implantitis.

Exosomes are known to provide an important advantage in periodontal therapy and even in the diagnosis of systemic diseases.^21^ Among them, long non-coding RNA (lncRNA) is a class of RNA molecules more than 200 nucleotides in length, which have no protein-coding function but can be involved in regulating cellular functions and disease processes. A recent study found that the abnormal expression of lncRNA MAFG-AS1 is associated with the occurrence and development of tumors.^14^ Huang et al^11^ claimed that knockdown of the lncRNA SNHG16 sponge miR-212-3p/NF-κB axis suppressed the diabetic inflammatory response. During the inflammatory process of peri-implantitis, the expression of some lncRNAs has also been confirmed to change and participate in the regulation of a variety of inflammatory pathways.^36^ X-inactive specific transcript (XIST) is a widely studied lncRNA that has been found to have therapeutic efficacy in cardiovascular diseases, immune disorders, and inflammatory reactions.^1,3^ Unfortunately, XIST has rarely been reported in the pathobiology of peri-implant diseases. Therefore, we hypothesised that XIST is abnormally elevated in patients with periimplantitis and contributes to early diagnosis.

In the context of existing research, this study determined the expression of salivary XIST in patients with peri-implantitis, and subsequently explored the diagnostic ability of XIST to identify patients with peri-implantitis and patients with healthy peri-implant tissues. Through the collection of general data of the patients, the relationship between abnormally expressed XIST and peri-implant indicators was analysed, and the association between XIST and disease progression was determined. Moreover, the possible regulatory mechanism of XIST in peri-implantitis was revealed based on the competitive binding relationship between lncRNA and microRNA (miRNA).

## MATERIALS AND METHODS

### Study Population

The statistical power was calculated using GPower software, version 3.1.9.2, to calculate the required sample size for the study. The effect size was set to 0.5, statistical significance was set at 0.05, and the statistical test power was 0.8. The results show that the research required in 128 participants.

The present study included 177 patients with implant restorations who were seen in the Department of Dentistry of The First Affiliated Hospital of Anhui Medical University from January 2023 to June 2023. The included patients were categorised into a healthy control group (n = 80) and a peri-implantitis group (n = 97) according to whether they had peri-implantitis or not. The inclusion requirements for patients with peri-implantitis were: (1) suffering from peri-implantitis according to the criteria established by the 2017 Workshop on Classification of Periodontal and Peri-implant Diseases and Conditions; (2) clinical symptoms: bleeding, suppuration, bone loss, alveolar bone loss, and increased pocket depth; (3) time of denture implantation was more than three months; (4) no occlusal trauma to the implant. The exclusion criteria were: (1) concomitant peri-implant diseases such as periodontitis and gingivitis; (2) patients undergoing orthodontic treatment; (3) patients with combined diabetes or tumors; (4) patients who had used antibiotics and immunosuppressants within three months prior to starting the study.

This study was performed in line with the principles of the Declaration of Helsinki. Approval was granted by the Ethics Committee of The First Affiliated Hospital of Anhui Medical University (Approval number: 20222017), and the included patients voluntarily participated after being informed of the purpose of the study.

### Sample Collection

Saliva samples were collected in the early morning under fasting conditions. Before collecting the samples, patients rinsed their mouths and did not swallow the saliva produced. The cotton mass was then placed in the oral cavity for 2-5 min from which saliva samples were taken. Saliva samples (1 ml) were stored in centrifuge tubes and centrifuged at 3000 r/min for 15 min at 4°C to obtain saliva supernatant.

### Detection of Peri-Implant Parameters

Oral examination was performed using a Williams peri-implant probe, and the plaque index (PLI), sulcus bleeding index (SBI) and probing depth (PD) of the included patients were recorded.

### RT-qPCR Assays

Total RNA in the samples was obtained by treatment with Trizol reagent (Invitrogen; Carlsbad, CA, USA). PrimeScript RT Kit (TaKaRa, Tokyo, Japan) and TaqMan microRNA Reverse Transcription Kit (Invitrogen) were selected to reverse transcribe RNA to cDNA. RT-qPCR assays were performed in an Applied Biosystems 7900 Real-Time PCR system (Applied Biosystems; Carlsbad, CA, USA), and the reaction system needed to be configured by the SYBR Premix Ex Taq kit (TaKaRa) before operation. GAPDH and U6 were used as internal controls for XIST and miR-150-5p, and their expression was calculated by the 2-ΔΔCt method, with three replicates performed for each group.

### Cell Culture

Human gingival fibroblasts (#2620) were purchased from Sciencell (Invitrogen). The above cells were transferred to DMEM medium supplemented with 10% FBS and 1% penicillin/streptomycin (Gibco; Rockville, MD, USA), and incubated in a 5% CO_2_ incubator at 37°C.

### Luciferase Reporter Gene Assay

The potential targets of XIST were predicted by the ENCORI, and further verified by luciferase activity assay. Wild-type (WT-XIST) and mutant-type (MT-XIST) reporter vectors were obtained by transferring the XIST fragment into the PGL3 vector. The gingival fibroblasts (4 x 10^5^) were cultured in 12-well plates, and cell transfection assay was performed. WT-XIST or MT-XIST was co-transfected with miR-150-5p mimic/inhibitor or miR-NC into cells by lipofectamine 3000 (Invitrogen). The miR-150-5p mimic/inhibitor and miR-NC were provided by GenePharma (Suzhou, China). After the transfection assay was performed for 48 h, the luciferase activity of the cells was verified using a dual luciferase reporter system (Promega; Madison, WI; USA).

### Statistical Analysis

The relevant clinical data were statistically analysed using GraphPad Prism 7.0 software. Count data were expressed as n, and measurement data were expressed as mean ± SD. Differences between the healthy control group and the peri-implantitis group were comparatively analysed using the Student t-test. The diagnostic potential of XIST for peri-implantitis was evaluated by ROC curve. Pearson correlation analysis identified the correlation between XIST expression and periodontological indicators. Logistic regression analysis was used to evaluate the effect of different variables on the occurrence of peri-implantitis.

## RESULTS

### Comparison of General Information of Included Patients

The general information of the included patients is recorded in Table 1. The analysis showed that there was no stastistically significant difference in age (p=0.334), sex (p=0.900), implant location (p=0.954), bleeding or suppuration (p=0.054), prosthetic design (p=0.068), brushing daily (p=0.207), smoking status (p=0.892) and alcohol consumption (p=0.907) between the healthy control and the peri-implantitis group. However, PLI, SBI and PD levels, probing pocket depth (p<0.001), clinical attachment loss (p=0.048), bleeding scores, plaque scores (p<0.001) were statistically significantly upregulated in patients with peri-implantitis. Meanwhile, the number of patients with a history of periodontal disease was higher (p=0.001), and the difference was statistically significant.

**Table 1:** The baseline data of the study subjects

Parameter	Healthy control (n=80)	peri-implantitis (n=97)	p-value
Age (years)	40.90±8.19	42.36±11.24	0.334
Sex			0.900
male	42	50	
female	38	47	
Plaque index (PLI; scores)	1.81±0.53	3.03±0.78	<0.001
Sulcus bleeding index (SBI; scores)	0.79±0.18	2.82±0.16	<0.001
Probing depth (PD; mm)	2.39±0.39	4.91±1.08	<0.001
Probing pocket depth (mm)	2.99±0.62	3.82±1.34	<0.001
Clinical attachment loss (mm)	4.40±0.85	4.75±1.35	0.048
Bleeding scores	0.51±0.04	1.51±0.47	<0.001
Plaque scores	0.64±0.28	1.81±0.77	<0.001
Implant location			0.954
Maxillary	49	59	
Mandibular	31	38	
Bleeding or suppuration			0.054
No	72	77	
Yes	8	20	
Periodontal disease history			0.001
No	56	44	
Yes	24	53	
Prosthetic design			0.068
Fixed	65	88	
Removable	15	9	
Brushing daily			0.207
1–3 times	73	93	
More than 3 times	7	4	
Smoking status			0.892
No	52	64	
Yes	28	33	
Alcohol consumption			0.907
No	43	53	
Yes	37	44	

### XIST Expression and Diagnostic Properties

**Fig 1:**
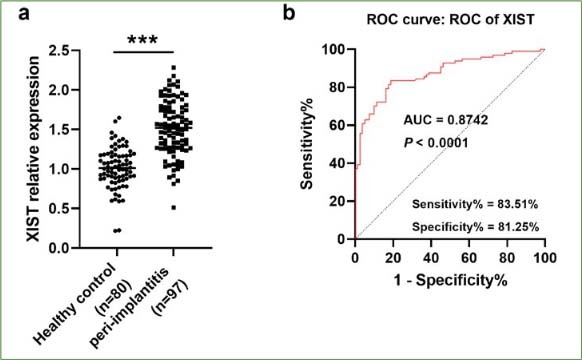
The detection and diagnostic value of XIST. (a) XIST expression was higher in patients in the peri-implantitis group than in the healthy control group (***p < 0.001). (b) ROC curve of XIST in the diagnosis of peri-implantitis (AUC = 0.8742, p < 0.001).

RT-qPCR assay revealed that the expression of XIST in patients with peri-implantitis (n=97) was elevated compared to the healthy control group (n=80) (Fig 1a). Furthermore, the sensitivity and specificity of XIST in distinguishing peri-implantitis patients were 83.51% and 81.25%, and the AUC was 0.8742 (Fig 1b, p < 0.001), suggesting that XIST has a high potential for the diagnosis of peri-implantitis.

Logistic regression analysis revealed that XIST (OR = 0.036, 95% CI = 0.265-4.600, p = 0.001), PLI (OR = 0.068, 95% CI = 0.017-0.321, p = 0.005), SBI (OR = 0.008, 95% CI = 0.002-0.060, p < 0.001), PD (OR = 0.018, 95% CI = 0.004-0.122, p < 0.001) levels and peri-implant disease history (OR = 0.091, 95% CI = 0.036-0.789, p = 0.018) may be related to the occurrence of peri-implantitis, which was the risk factor of peri-implantitis (Table 2).

**Table 2:** Logistic regression analysis of clinical variables in patients with peri-implantitis

Items	OR	95% CI	p-value
XIST	0.036	0.265 – 4.600	0.001
Age (years)	1.103	0.265–4.600	0.909
Sex	1.245	0.319–6.221	0.811
Plaque index (PLI)	0.068	0.017–0.321	0.005
Sulcus bleeding index (SBI)	0.008	0.002–0.060	<0.001
Probing depth (PD)	0.018	0.004–0.122	<0.001
Implant location	1.909	0.329–5.332	0.507
Bleeding or suppuration	1.732	0.048–2.818	0.668
Periodontal disease history	0.091	0.036–0.789	0.018
Prosthetic design	0.461	0.044–3.210	0.547
Brushing daily	3.021	0.071–8.276	0.433
Smoking status	0.431	0.094–1.780	0.328
Alcohol consumption	2.993	0.071–1.687	0.259

### Correlation Between XIST Expression and Peri-Implant Parameters

**Fig 2:**
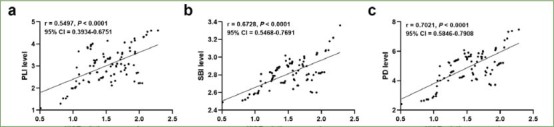
Relationship between XIST expression and peri-implant indicators. XIST expression was positively correlated with the levels of PLI (a; r = 0.5497, p < 0.0001), SBI (b; r = 0.6728, p < 0.0001) and PD (c; r = 0.7021, p < 0.0001).

The relationship between XIST levels and peri-implant parameters was evaluated presented using Pearson’s correlation coefficient. Figure 2a demonstrates that PLI levels were positively correlated with XIST expression (r = 0.5497, p < 0.0001, 95% CI = 0.3934-0.6751), and similarly, SBI (Fig 2b; r = 0.6728, p < 0.0001, 95% CI = 0.5468-0.7691) and PD (Fig 2c; r = 0.7021, p < 0.0001, 95% CI = 0.5846-0.7908) expression were also proportional to XIST expression, implying that XIST expression was associated with disease severity.

### XIST and Downstream Target miR-150-5p

**Fig 3:**
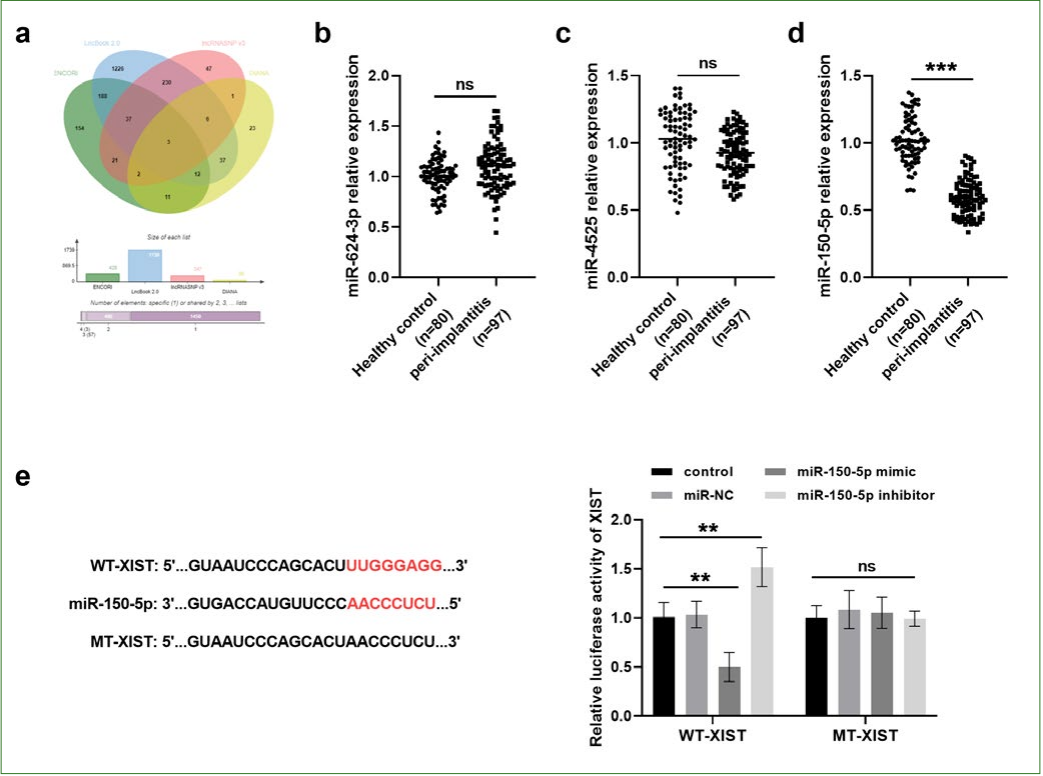
XIST directly targets downstream miR-150-5p. (a) Venn diagram of potential downstream targets of XIST. Expression of miR-624-3p (b), miR-4525 (c) and miR-150-5p (d) was examined in peri-implantitis group and healthy control group (ns p > 0.05, ***p < 0.001). (e) The targeting relationship between XIST and miR-150-5p was assessed by bioinformatics prediction and luciferase activity assay (ns p > 0.05, **p < 0.01).

The possible downstream targets of XIST were predicted by online biology websites (ENCORI, LncBook 2.0, lncRNASNP v3, DIANA), and a Venn diagram was drawn (Fig 3a). The relative expression levels of miR-624-3p, miR-4525 and miR-150-5p were verified in the healthy control group and peri-implantitis group, and miR-150-5p was found to be downregulated in the peri-implantitis group (Fig 3b-d). Furthermore, the complementary sequences of XIST and miR-150-5p were queried by the online database ENCORI (Fig 3e). The luciferase reporter gene assay confirmed that transfection of miR-150-5p mimic decreased luciferase activity in the WT-XIST group, whereas transfection of miR-150-5p inhibitor gave the opposite experimental results. However, transfection with miR-150-5p mimic/inhibitor did not statistically significantly alter luciferase activity in the MT- XIST group. It is thus understood that XIST negatively regulates the level of miR-150-5p (Fig 3e).

## DISCUSSION

Peri-implantitis is an inflammatory reaction of the soft and bony tissues surrounding the implant, leading to loosening or detachment of the implant.^27^ In modern medical practice, antibiotic treatment, surgical treatment or implant removal treatment are determined according to the specific condition of the patient.^2^ However, regardless of the type of treatment chosen, early detection and intervention can improve the outcome and reduce the occurrence of complications.

XIST is a non-coding RNA gene located on the X chromosome with a length of 17kb, and is involved in the process of X chromosome inactivation.^25^ Either mutation or overexpression of XIST may lead to abnormalities in the non-activating processes of the X chromosome, which in turn may induce a range of diseases.^24^ For example, XIST has been identified to mediate tumor progression in colorectal cancer, breast cancer, ovarian cancer, and liver cancer.^16,17,20,34^ More fundamentally, Feng et al^9^ noted that XIST targeting miR-214-3p affects the growth of peri-implant ligament stem cells. Xu et al^32^ also described the regulatory role of the XIST/USF2/WDR72 axis in the development of periodontitis. In this study, XIST was verified to be upregulated in patients with peri-implantitis. Furthermore, XIST was able to distinguish peri-implantitis patients from controls, and logistic regression analysis elucidated the potential of XIST as a risk factor for peri-implantitis.

The periodontal indicators PLI, SBI and PD are objective indicators for assessing the status of oral hygiene and periodontal inflammation.^10^ PLI represents the degree of plaque accumulation on the dental surface, and thus reflects the patient’s oral hygiene status. The increased PLI value indicates more plaque accumulation on the dental surface and increases the risk of peri-implantitis.^23^ SBI is used to assess bleeding from the peri-implant tissues, and high SBI values indicate a higher degree of inflammation there, which may be associated with the development of peri-implantitis.^8^ PD is an indicator of the depth of the periodontal pockets; larger PD values increase the risk of developing periodontal inflammation.^33^ Through clinical index analysis, we found that the periodontal indicators PLI, SBI, and PD levels were elevated in patients with peri-implantitis, and Pearson’s correlation analysis showed that PLI, SBI, and PD were positively correlated with XIST expression, suggesting that the XIST expression was related to the severity of the disease.

miR-150-5p is located on chromosome 19q13.33 and is dysregulated in several cancers.^26^ We also confirmed that miR-150-5p expression was lower in peri-implantitis patients than in healthy control via qRT-PCR assays. Meanwhile, as predicted by the biological online website and elaborated by the luciferase reporter gene assay, miR-150-5p is a direct target of XIST in this study, which was consistent with the findings of Wang et al.^29^ Wei et al^30^ revealed that the expression of miR-150-5p decreased in sepsis cell samples and was involved in sepsis inflammatory response. In addition, there is evidence that miR-150-5p was downregulated in peri-implantitis and was related to the occurrence of peri-implantitis.^6^ Interestingly, most chronic inflammatory diseases are associated with excessive production and abnormal regulation of inflammatory factors.^13^ For example, Li et al^15^ claimed that the continuous release of inflammatory factors can damage peri-implant tissues, which may cause the appearance of peri-implantitis and ultimately lead to implant failure. Based on this, XIST may be involved in the process of peri-implantitis through sponge miR-150-5p; the molecular mechanism and the inflammatory factors involved are targets of our future research.

By measuring and analysing the content of XIST in saliva samples of patients, we revealed that prominently expressed XIST has a high diagnostic value in early peri-implantitis, providing a new reference for the treatment of patients and the study of related molecular mechanisms. However, the limited number of samples included in the experiment may have potentially biased the results. In addition, the lack of cell experimental design limited the breadth and depth of this research. The above problems need to be addressed and further explored in subsequent studies.

## CONCLUSION

Salivary XIST is highly expressed in patients with peri-implantitis and is associated with the severity of the condition. Meanwhile, XIST may be a diagnostic marker for peri-implantitis with positive clinical potential in the prevention and treatment of patients.
